# Gemcitabine triggers angiogenesis-promoting molecular signals in pancreatic cancer cells: Therapeutic implications

**DOI:** 10.18632/oncotarget.3784

**Published:** 2015-04-23

**Authors:** Mohammad Aslam Khan, Sanjeev K. Srivastava, Arun Bhardwaj, Seema Singh, Sumit Arora, Haseeb Zubair, James E. Carter, Ajay P. Singh

**Affiliations:** ^1^ Department of Oncologic Sciences, Mitchell Cancer Institute, University of South Alabama, Mobile, Alabama, USA; ^2^ Department of Pathology, College of Medicine, University of South Alabama, Mobile, Alabama, USA; ^3^ Department of Biochemistry and Molecular Biology, College of Medicine, University of South Alabama, Mobile, Alabama, USA

**Keywords:** pancreatic cancer, gemcitabine, angiogenesis, IL-8

## Abstract

Pancreatic tumor microenvironment (TME) is characterized by poor tumor-vasculature and extensive desmoplasia that together contribute to poor response to chemotherapy. It was recently shown that targeting of TME to inhibit desmoplasiatic reaction in a preclinical model resulted in increased microvessel-density and intratumoral drug concentration, leading to improved therapeutic response. This approach; however, failed to generate a favorable response in clinical trial. In that regard, we have previously demonstrated a role of gemcitabine-induced CXCR4 signaling as a counter-defense mechanism, which also promoted invasiveness of pancreatic cancer (PC) cells. Here, we investigated the effect of gemcitabine on endothelial cell phenotype. Gemcitabine-treatment of human-umbilical-vein-endothelial-cells (HUVECs) did not promote the growth of HUVECs; however, it was induced when treated with conditioned media from gemcitabine-treated (Gem-CM) PC cells due to increased cell-cycle progression and apoptotic-resistance. Moreover, treatment of HUVECs with Gem-CM resulted in capillary-like structure (CLS) formation and promoted their ability to migrate and invade through extracellular-matrix. Gemcitabine-treatment of PC cells induced expression of various growth factors/cytokines, including IL-8, which exhibited greatest upregulation. Further, IL-8 depletion in Gem-CM diminished its potency to promote angiogenic phenotypes. Together, these findings suggest an indirect effect of gemcitabine on angiogenesis, which, in light of our previous observations, may hold important clinical significance.

## INTRODUCTION

Pancreatic cancer (PC) is largely an incurable malignancy and one of the deadliest cancers in the United States. According to American Cancer Society, approximately 48,960 Americans will be diagnosed with PC in 2015 and it will claim nearly 40,560 lives [1]. The median survival after diagnosis is ~2–8 months, and only ~6% of all patients with PC survive 5 years post-diagnosis [2]. At the time of diagnosis, most pancreatic tumors are either highly genetically advanced or have spread to distant sites leaving systemic chemotherapy as the only viable option for treatment [3]. Disappointingly, none of the chemotherapeutic regimen (single agent or combination) has been very successful, and all provide only marginal survival benefit at the best to the PC patients [4, 5]. Moreover, there also remains a concern for unintended and undesired effects of chemotherapy supported by recent findings [3, 6]. Therefore, it is extremely important that we develop an improved understanding of the mechanisms underlying chemoresistance of PC as well as the host response to chemotherapy that may adversely affect overall clinical outcome.

Angiogenesis is a process of new blood vessel formation from the existing ones and plays important role in tumor progression and metastasis [7, 8]. Remarkably, pancreatic tumors are poorly vascularized relative to other solid tumor types, and this unusual phenotype is considered one of the main reasons underlying their aggressive behavior and therapy-resistance [9, 10]. In a preclinical study targeted at improving the therapeutic outcome of gemcitabine in PC, depletion of tumor-associated stroma through hedgehog inhibition promoted drug accumulation and associated with an increase in tumor vascularization [11]. Although increased vasculature was suggested to promote drug delivery at the tumor site, mechanism(s) underlying this process remained unanswered. Furthermore, true significance of this induced phenotype is also debatable as the similar therapeutic approach failed in a clinical trial [12]. It is likely that the increased tumor vasculature may work both ways i.e. increase drug delivery to the tumor site or facilitate escape of the tumor cells to other niches favoring tumor cell survival and thus chemoresistance. Latter seems further plausible considering our recent observation, where treatment of PC cells with gemcitabine promoted their invasiveness through upregulation of CXCR4 [3]. Chemotherapy-induced angiogenesis has been reported in other cancer as well [13]. Moreover, it has also been shown that drug treatment leads to an acute recruitment of circulating endothelial progenitor cells to tumors in mice [14].

The present study was undertaken to examine the effect of gemcitabine on endothelial cell phenotype and identify potential mediator(s) in this process. Our data demonstrate that gemcitabine does not have a direct favorable impact on endothelial cells, but rather affects them via inducing angiogenic signals in treated PC cells. We show gemcitabine induces the expression of several angiogenesis-promoting cytokines with highest upregulation in the levels of IL-8. Depletion of IL-8 from conditioned media of gemcitabine-treated PC cells diminished its promotion of endothelial cell proliferation, survival, motility and invasiveness, and capillary formation. Together, these findings shed new light on another unintended effect of chemotherapy, whose clinical significance remains yet to be established.

## RESULTS

### Conditioned media from gemcitabine-treated pancreatic cancer cells enhances growth of endothelial cells by promoting cell-cycle progression and survival

**Figure 1:**
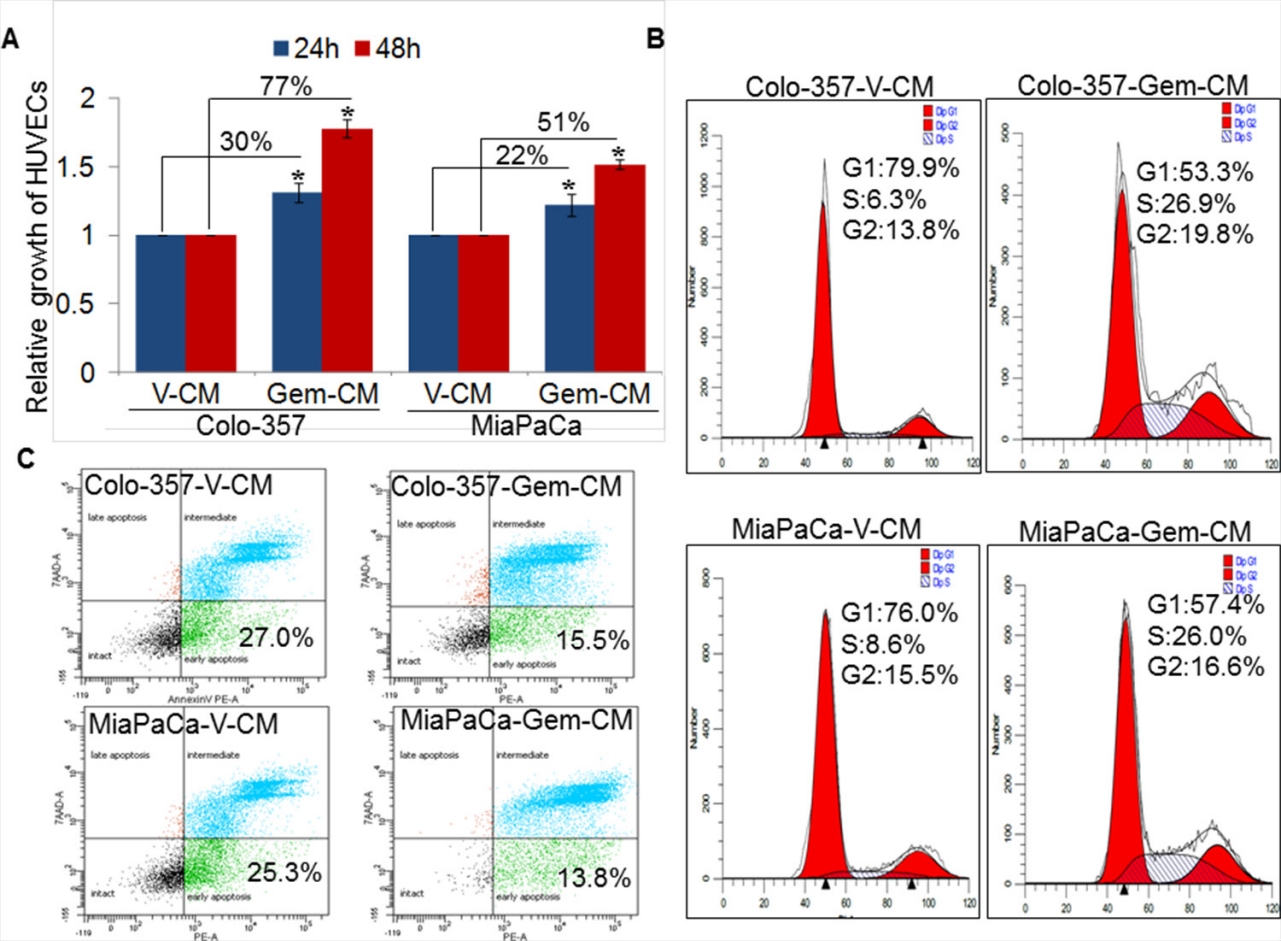
Effect of conditioned media obtained from gemcitabine- or vehicle- treated pancreatic cancer cells on endothelial cell growth, cell-cycle progression and survival **A.** HUVECs (1 × 10^4^ cells/well) were seeded in 96-well plates and allowed to grow for 24 h followed by treatment with conditioned media (CM) obtained from vehicle (V-CM) or gemcitabine (Gem-CM) treated PC (Colo-357 and MiaPaCa) cells. Growth of HUVECs was measured by WST-1 assay after 24 h and 48 h of incubation in CM. Bars (mean ± SD; *n* = 3) represent fold change in growth. *, *p* < 0.05. **B.** Synchronized HUVECs were treated with V-CM or Gem-CM for 24 h and distribution of cells in different phases of cell cycle was analyzed by propidium iodide (PI) staining through flow cytometry. **C.** HUVECs (1 × 10^6^) were grown in 6-well plate for 24 h, treated with V-CM or Gem-CM for next 48 h, and subsequently stained with 7-AAD and PE Annexin V followed by flow cytometry.

Since enhanced gemcitabine accumulation at the tumor site coincided with enhanced blood vessel formation [11], we first examined the effect of gemcitabine treatment on the growth of human umbilical vein endothelial cells (HUVECs). The data show no change or a decreased (at high doses) growth of HUVECs upon treatment with gemcitabine (Supplementary Figure 1). Therefore, we next examined the indirect effect of gemcitabine on endothelial cell growth by treating them with the conditioned media from vehicle (V-CM) or gemcitabine (Gem-CM) treated PC (Colo-357 and MiaPaCa) cells for 24 and 48 h. Our data reveal a significant increase in the growth of HUVECs when treated with Gem-CM obtained from Colo-357 and MiaPaCa cells. The growth of HUVECs is increased by ~30 % and ~22 % upon 24 h treatment of Gem-CM from Colo-357 and MiaPaCa cells, respectively, as compared with the HUVECs treated with respective V-CM (Figure 1A). This difference grew further at 48 h with ~77 % and ~51 % increase in the growth of HUVECs, when treated with Gem-CM from Colo-357 and MiaPaCa cells, respectively.

We next examined the effect of Gem-CM on cell cycle progression and survival of endothelial cells. Our cell-cycle data demonstrate an enhanced cell-cycle progression in HUVECs treated with Gem-CM. A greater fraction (~26.9 % and ~26 %) of HUVECs is detected in S-phase upon treatment with Colo-357-Gem-CM and MiaPaCa-Gem-CM, respectively as compared to those treated with Colo-357-V-CM (~6.3 %) and MiaPaCa-V-CM (~8.6 %) (Figure 1B). In addition, the data from apoptosis assay indicate lower apoptotic index in HUVECs treated with Colo-357-Gem-CM (~15.5 %) and MiaPaCa-Gem-CM (~13.8 %) in comparison to those treated with V-CM (~27 %) from Colo-357 and MiaPaCa (~25.3 %) (Figure 1C). Together, these findings indicate that Gem-CM enhances growth of endothelial cells by promoting cell-cycle progression and apoptosis resistance.

### Conditioned media from gemcitabine-treated pancreatic cancer cells promotes *in vitro* angiogenesis and migration and invasion of endothelial cells

**Figure 2:**
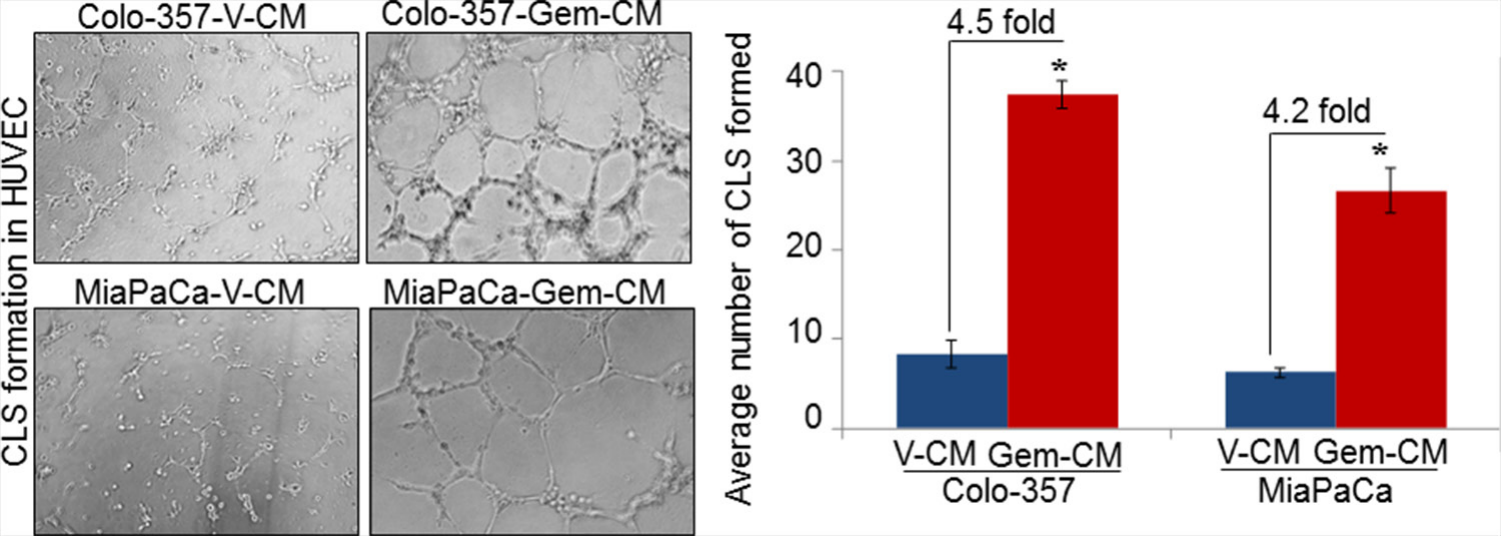
Conditioned media from gemcitabine-treated pancreatic cancer cells facilitates capillary-like structure (CLS) formation in HUVEC HUVECs (1 × 10^4^) were plated on Matrigel-coated 96-well plates in conditioned media (CM) obtained from vehicle (V-CM) or gemcitabine (Gem-CM) treated Colo-357 and MiaPaCa cells. After 16 h of incubation, CLS formation was examined under inverted microscope, photographed and number of CLS formation counted in 10 random fields. Bars (mean ± SD; *n* = 3) represent number of CLS per fields. *, *p* < 0.05.

Having observed growth induction of endothelial cells upon treatment with conditioned media from gemcitabine-treated (Gem-CM) PC cells, we next examined if Gem-CM would also promote the *in vitro* angiogenesis. For this, HUVECs were seeded in Matrigel-coated 96-well plate in the presence of V-CM or Gem-CM for 16 h and effect on the capillary-like structure (CLS) formation was examined. Our data demonstrate that treatment of HUVECs with Gem-CM resulted in robust CLS formation (Figure 2). HUVECs treated with Colo-357-Gem-CM and MiaPaCa-Gem-CM exhibit enhanced number of CLS (~38 and ~29, respectively) as compared to those treated with Colo-357-V-CM (~8) and MiaPaCa-V-CM (~6) (Figure 2).

**Figure 3:**
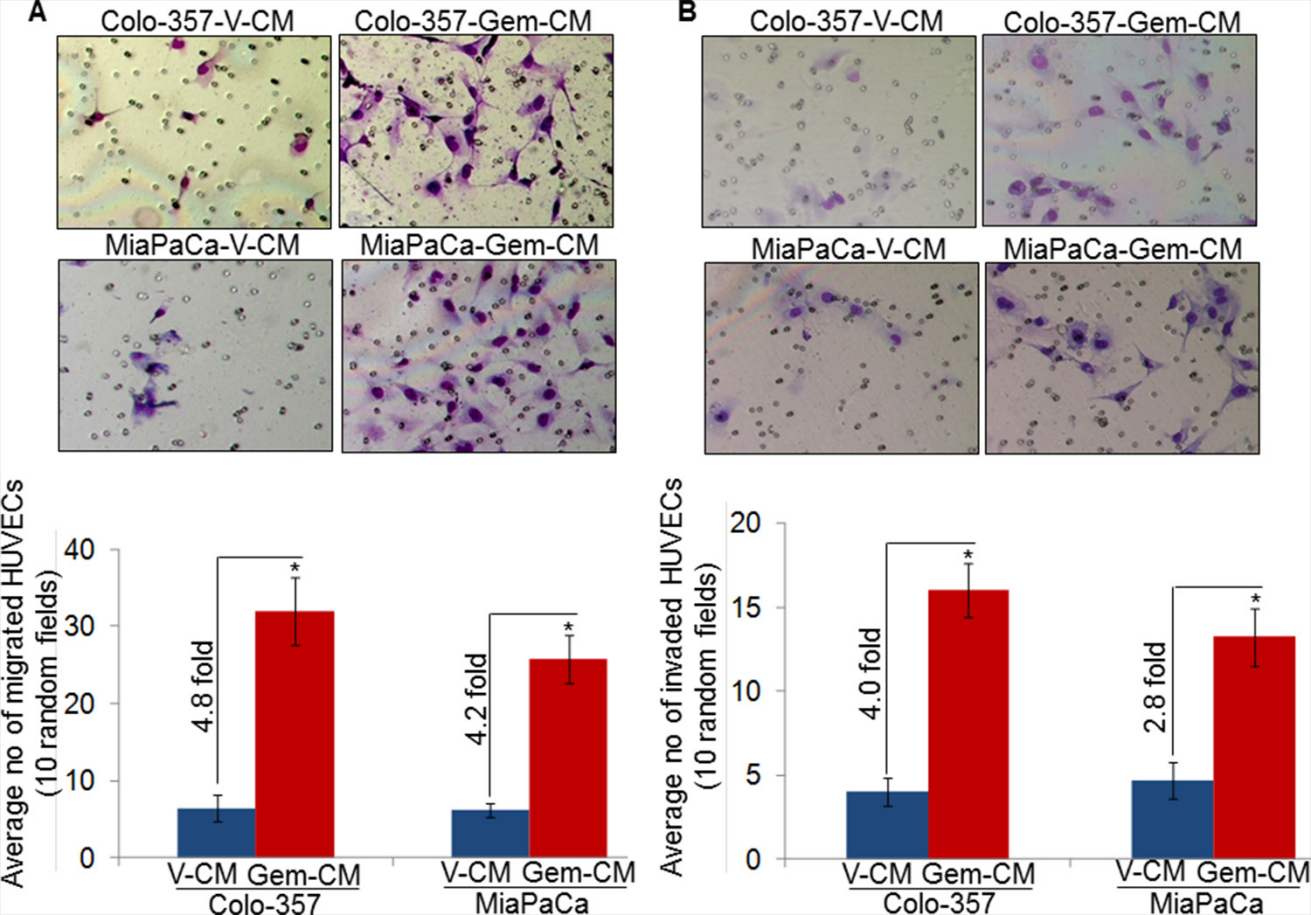
Conditioned media from gemcitabine-treated pancreatic cancer cells promotes motility and invasion of endothelial cells HUVECs were seeded on **A.** non-coated (for motility assay), or **B.** Matrigel-coated (for invasion assay) membranes. V-CM or Gem-CM obtained from Colo-357 and MiaPaCa were used as a chemoattractant. Migrated and invaded cells were counted and presented as average number of cells in 10 random field ± SD. Data is representative of three independent experiments.*, *p* < 0.05.

Migratory and invasive potential of endothelial cells is indispensable for angiogenesis [15]. Therefore, we next examined the effect of Gem-CM from PC cells on the migration and invasion of HUVECs. For this, HUVECs cells were seeded in the top chamber of non-coated or Matrigel-coated membrane inserts in serum-free media and V-CM or Gem-CM from PC cells were used as chemoattractant. The data show a significantly greater motility of HUVECs (~4.8 and ~4.2 folds, respectively), when Gem-CM from Colo-357 and MiaPaCa cells is used as a chemoattractant in comparison to that from vehicle-treated (V-CM) PC cells (Figure 3A). Similarly, greater number of HUVECs (~4.0 and ~2.8 folds) invaded through the Matrigel barrier in presence of Gem-CM from Colo-357 and MiaPaCa, respectively, as compared to that from V-CM (Figure 3B). Importantly, when we pre-treated HUVECs for 12 h with V-CM or Gem-CM, a greater effect of Gem-CM on motility and invasion of HUVECs was recorded (Supplementary Figure 2). Collectively, our findings suggest that Gem-CM has the potential to trigger angiogenic phenotype in endothelial cells.

### Gemcitabine induces expression of angiogenesis-associated cytokines in pancreatic cancer cells

**Figure 4:**
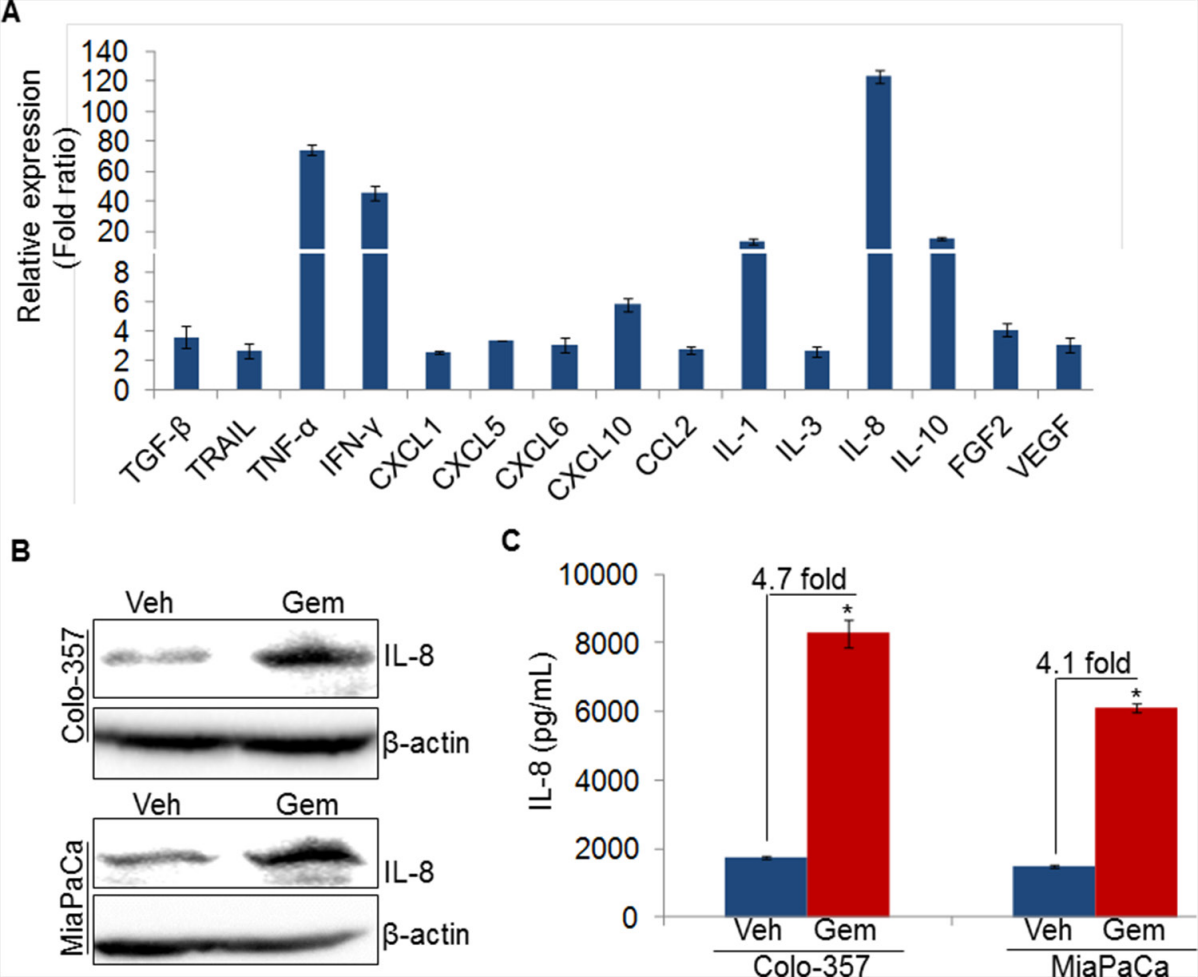
Gemcitabine induces IL-8 expression in pancreatic cancer cells **A.** Colo-357 cells were treated with gemcitabine (10 μM) for 8 h. Subsequently, RNA was isolated, cDNA was prepared and cytokines/growth factors profiling was performed using qRT-PCR. **B.** Colo-357 and MiaPaCa cells were treated with gemcitabine (10 μM) for 8 h. Post treatment, media was replaced with fresh culture medium and incubated for next 24 h. Thereafter, total protein was isolated and subjected to immunoblot analysis to examine IL-8 expression using specific antibody. β-actin was used as a loading control. **C.** Level of IL-8 in conditioned media of vehicle or gemcitabine treated PC cells was measured using ELISA as described in materials and methods. Data is presented as mean ± SD; *n* = 3 .*p < 0.05.

Cytokines or growth factors secreted by tumor cells play important roles in the endothelial cell proliferation and new blood vessels formation at tumor site [8, 16, 17]. To understand the molecular mechanism of the Gem-CM-induced angiogenesis, we treated PC (Colo-357) cells with vehicle or gemcitabine for 8 h, and effect on the various angiogenesis-associated cytokines and/or growth factors was examined by quantitative RT-PCR. Our data demonstrate that among the 25 genes analyzed (Supplementary Table 1), we observed 15 cytokines/growth factors to be up-regulated (≥ two fold difference; *p* value ≤ 0.05) in gemcitabine-treated Colo-357 cells (Figure 4A). Interestingly, we observed the highest induction in the expression of IL-8 (~123 fold), which is secreted by pancreatic tumor cells and known to trigger angiogenesis through the recruitment of immune cells at tumor site [15, 17]. To validate the IL-8 induction in gemcitabine treated PC cells, Colo-357 and MiaPaCa cells were treated with vehicle or gemcitabine and effect on IL-8 at protein level was examined by immunoblot analysis. We observed enhanced expression of IL-8 in both the PC cells upon gemcitabine treatment as compared to vehicle treated PC cells (Figure 4B). Moreover, the amount of secreted IL-8 by the Colo-357 and MiaPaCa cells following gemcitabine treatment was also determined by ELISA. Data show that level of IL-8 is increased in the culture supernatant of gemcitabine-treated Colo-357 (~4.7 fold) as well as MiaPaCa (~4.1 fold) cells as compared to their respective vehicle-treated controls (Figure 4C). Taken together, our data suggest that treatment of gemcitabine induces the expression of various cytokines including IL-8 in PC cells.

### IL-8 inhibition decreases Gem-CM-induced effects on endothelial cells

**Figure 5:**
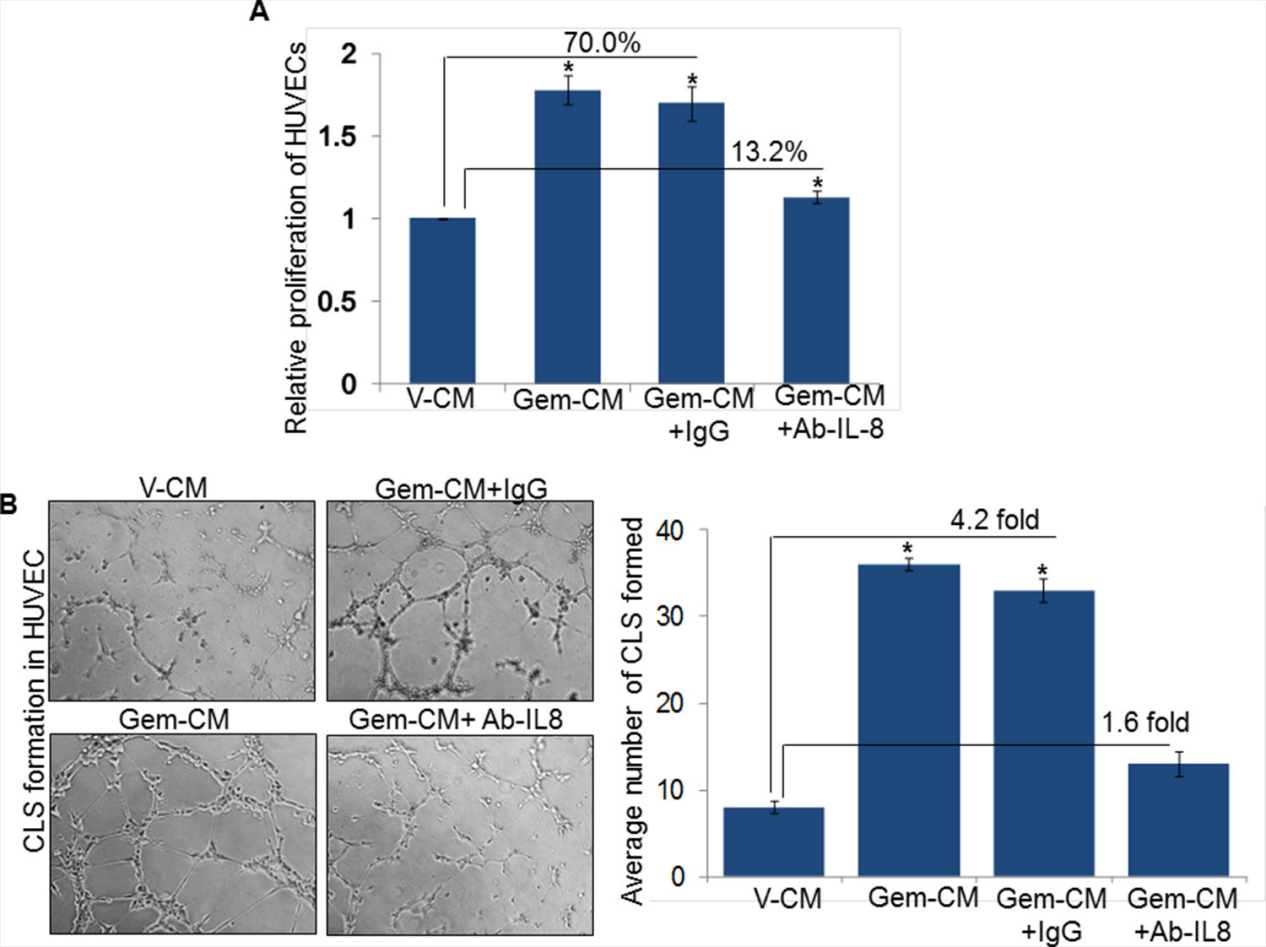
Neutralization of IL-8 abolishes Gem-CM-induced HUVEC proliferation and CLS formation **A.** HUVECs (1 × 10^4^ cells/well) were seeded in 96-well plates, treated with V-CM, Gem-CM or Gem-CM pre-treated with IL-8 neutralizing antibody or control IgG (200 ng/mL) and growth was measured by WST-1 assay after 48 h of incubation. **B.** HUVECs (1 × 10^4^) were plated on Matrigel-coated 96-well plates in V-CM, Gem-CM or Gem-CM pre-treated with IL-8 neutralizing antibody or control IgG. After 16 h of incubation, CLS formation was examined under inverted microscope, photographed and number of CLS formation counted in 10 random fields. Data is presented as mean ± SD; *n* = 3. *, *p* < 0.05.

**Figure 6:**
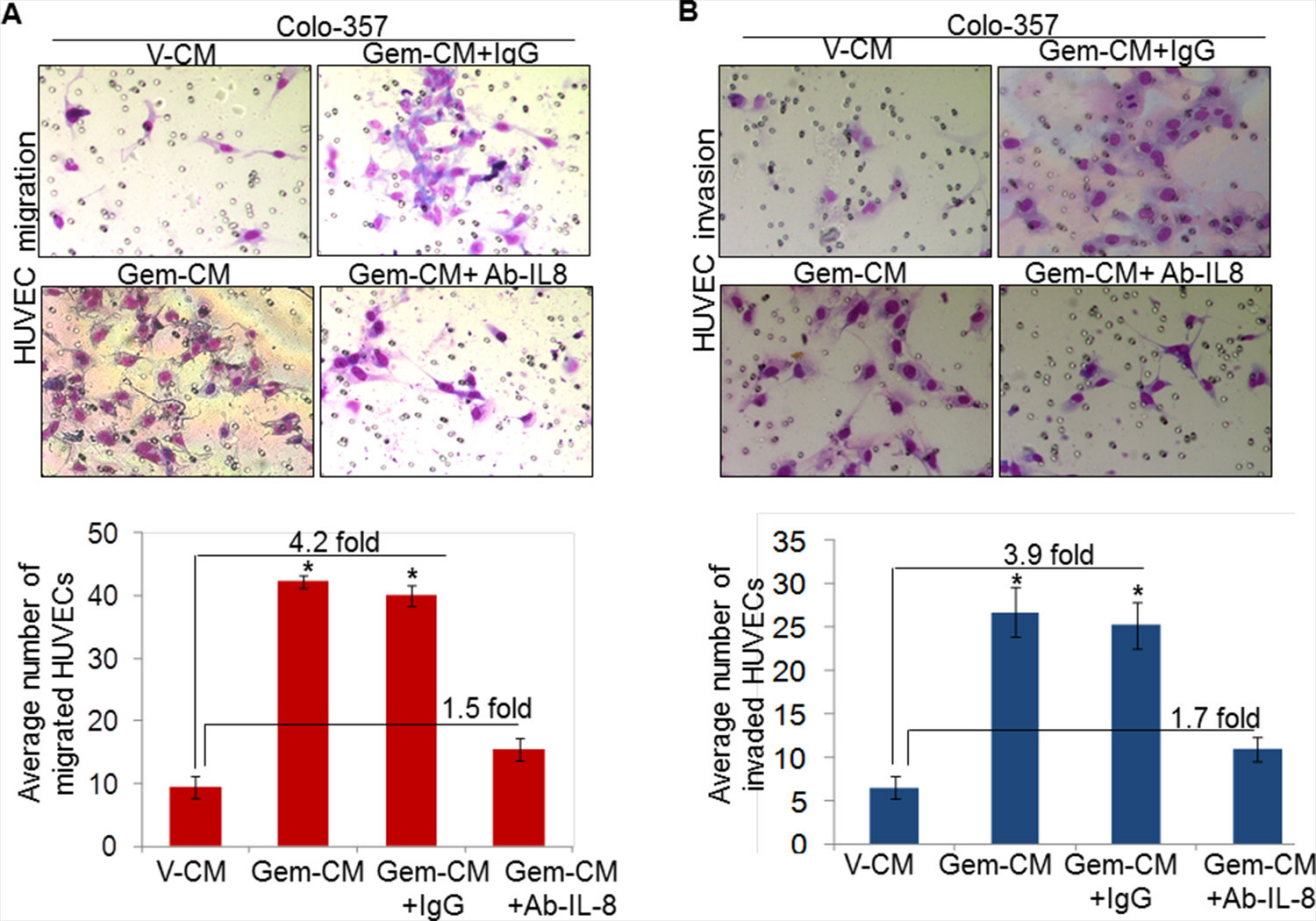
Depletion of IL-8 in Gem-CM decreases motility and invasiveness of HUVEC HUVECs were seeded on **A.** non-coated (for motility assay), or **B.** Matrigel-coated (for invasion assay) membranes. V-CM, Gem-CM or IL-8 depleted Gem-CM were used as chemoattractant. Bars represent mean ± SD (*n* = 3) of number of migrated or invaded cells per field. *, *p* < 0.05.

Having observed the induction of IL-8 following treatment of PC cells with gemcitabine, we next explored the involvement of IL-8 in Gem-CM promoted endothelial cell phenotypes. For this, Gem-CM form Colo-357 cells was pre-incubated with human IL-8 neutralizing antibody or control IgG for 24 h and effect on the phenotypes of HUVEC monitored. As expected, we observed significant growth induction (~70 %) of HUVECs by the Gem-CM pre-treated with control IgG (Figure 5A). Notably, very marginal growth-induction (~13.2 %) is observed when HUVECs were incubated with Gem-CM-pretreated with human IL-8 neutralizing antibody (Figure 5A). To validate a role of IL-8 in the Gem-CM-induced CLS formation, HUVECs plated on matrigel were treated with Gem-CM pretreated with control IgG or IL-8 neutralizing antibody. Data demonstrate that pretreatment of IL-8 neutralizing antibody significantly abrogates the Gem-CM-induced CLS formation in HUVEC cells as compared to Gem-CM pretreated with control IgG (Figure 5B). Similarly, our data from migration and invasion assay demonstrate that Gem-CM-induced effect on HUVEC migration (Figure 6A) and invasion (Figure 6B) was significantly inhibited upon neutralization of IL-8 in that. Together, these findings confirm that gemcitabine triggers the expression of IL-8 in PC cells that induces endothelial cells proliferation, CLS formation and increases motility and invasiveness.

## DISCUSSION

Angiogenesis is a fundamental phenomenon associated with the development, progression and therapy-resistance in almost every type of cancer including PC [8, 18]. It promotes the growth of tumor cells by nourishing them with oxygen and nutrients. In addition, it also enhances the metastasis by helping the spread of cancer cells throughout the body at their favorable niches [7, 19]. Successful angiogenesis is a multi-step process, which includes growth, invasion and migration, and differentiation of endothelial cells. The process usually begins when transformed cells begin secreting stromal modifying proteins, including angiogenic factors, and later may involve synergistic cooperation between tumor and stromal cells [7, 20–22]. The data presented herein indicate that this process is facilitated by the treatment of PC cells with gemcitabine through altered expression of angiogenic proteins. This effect is in contrast to an earlier report, which demonstrated that direct treatment of endothelial cells with anti-cancer drugs (doxorubicin, cisplatin and vincristine) induced angiogenic phenotypes [13]. In fact, we did not observe any growth promoting effect of gemcitabine on endothelial cells, but slightly decreased growth at high doses further suggesting that the effect of chemotherapy on angiogenesis promotion is rather indirect. Controlled cell cycle progression is critical for the development of new vasculature and elongation of the new capillary vessel [23]. Similarly, increased resistance to apoptosis in endothelial cells is also crucial during angiogenesis [24]. Directed migration and invasion, and formation of new capillary like structures are other important phenotypic behavior essential for angiogenesis [15, 25]. In corroboration with these notions, our data showed that Gem-CM not only promoted cell-cycle progression and survival of endothelial cells, but also enhanced their motility and invasive potential and capillary vessel formation.

Tumor-associated angiogenesis is tightly regulated by a fine balance of pro and anti-angiogenic factors including growth factors and/or cytokines present at tumor site [16, 26]. Pro-angiogenic effects of CM of gemcitabine-treated PC cells suggested that gemcitabine treatment programmed them for the secretion of angiogenesis-promoting factors. This notion was later supported by our data demonstrating gemcitabine-induced upregulation of the expression of various pro-angiogenic cytokines/growth factors, including IL-8. Moreover, pre-treatment of Gem-CM with IL-8 neutralizing antibody partially abolished the Gem-CM-induced phenotypes in endothelial cells confirming a participatory role of IL-8 in Gem-CM-promoted angiogenic effects. IL-8 is a pro-inflammatory cytokine secreted by variety of cancer cells including PC cells [27–29] and has been shown to positively affect tumor as well as endothelial cells through autocrine and paracrine signaling [15, 24, 29, 30]. Exogenous addition of recombinant IL-8 resulted in endothelial cell survival, proliferation and induced capillary formation, an effect which was abolished by inhibiting IL-8 [24]. IL-8 confers its effects on cellular phenotypes upon binding to its two cell surface cognate receptors, CXCR1 and CXCR2, which are widely expressed on the cancer as well as endothelial cells [31]. Consequently, silencing of these receptors has also been shown to inhibit tumor and/or endothelial cell proliferation, migration and capillary formation ability [31, 32]. Besides IL-8, we also observed significant induction of some other pro-angiogenic cytokines/growth factors, such as TNF-alpha upon gemcitabine treatment. However, previous studies suggest that TNF-alpha-induced neovascularization is mostly indirect. In the rabbit cornea, TNF-alpha induced angiogenesis in an IL-8 -dependent manner suggesting the molecular interaction between these two pathways [17]. Similarly, TNF-alpha promoted neovascularization in dental pulp cells through VEGF and SIRT1 [33]. Therefore, it appears that IL-8 plays a major role in gemcitabine-induced angiogenesis; however, its cooperative action with other angiogenic factors needs to be further examined.

Like many other tumor-promoting genes, expression of IL-8 in cancer cells can be regulated by a variety of mechanisms [34–36]. Our data show that gemcitabine induces IL-8 expression at transcriptional level in PC cells. Previous studies suggest IL-8 to be a potential target of NF-κB and HIF-1α [37, 38], which are associated with PC progression [2, 39]. Recently, we have shown that gemcitabine promotes the transcriptional activity of NF-κB and HIF-1α in PC cells by enhancing their nuclear level. Moreover, we also showed that effect of gemcitabine on NF-κB and HIF-1α is mediated through reactive oxygen species (ROS) generation in PC cells. These studies suggest a plausible mechanism for gemcitabine-induced IL-8 upregulation in PC cells via activation of NF-κB and/or HIF-1α. TNF-alpha is also reported to regulate IL-8 production through NF-κB [40]. Since we also identified TNF-alpha to be significantly upregulated in response to gemcitabine, it is possible that gemcitabine-mediated IL-8 upregulation is through both direct and indirect activation of NF-κB, and/or involves additional cross-talking mechanisms.

In conclusion, we have demonstrated that the gemcitabine treatment in PC cells results in the induction of IL-8, which in turn act on endothelial cells to promote their growth, migration and angiogenesis. These novel findings thus suggest that gemcitabine could induce the vasculature to the tumor site, which may either support resistance to chemotherapy by providing a more favorable growth environment or facilitate escape of tumor cells to other safe and chemoresistant niches.

## MATERIALS AND METHODS

### Cell lines and culture conditions

Colo-357 and MiaPaCa cells were procured and maintained as described earlier [22]. Human Umbilical Vein Endothelial Cells (HUVECs) were maintained in F12K medium supplemented with 0.1 mg/ml heparin, 0.03–0.05 mg/ml endothelial cell growth supplement (ECGS), 20 % fetal bovine serum (FBS), penicillin (100 units/mL) and streptomycin (100 μg/mL) at 37°C in a humidified atmosphere of 5 % CO_2_. All cells were regularly monitored for their typical morphology and intermittently tested for mycoplasma contamination at our Institutional facility.

### Reagents and antibodies

Dulbecco's Modified Eagle Medium (DMEM), Roswell Park Memorial Institute Medium (RPMI-1640), Kaighn's Modification of Ham's F-12 Medium (F12K), penicillin-streptomycin (Invitrogen, Carlsbad, CA); Fetal bovine serum (FBS) (Atlanta Biologicals, Lawrenceville, GA); WST-1 proliferation assay kit (Roche, Indianapolis, IN); High-Capacity RNA-to-cDNA™ Kit and SYBR Green Master Mix (Applied Biosystems, Carlsbad, CA); Diff-Quick cell staining kit (Dade Behring, Inc., Newark, DE); *In vitro* Angiogenesis kit (EMD Millipore, Temecula, CA); anti-human IL-8 ELISA Kit (R&D Systems Inc., Minneapolis, MN); Gemcitabine (Sigma-Aldrich, St. Louis MO); Western blotting SuperSignal West Femto Maximum sensitivity substrate kit (Thermo Scientific, Logan, UT); goat anti-IL-8 antibody (Abcam, Cambridge, MA); biotinylated anti-β-actin (Sigma-Aldrich) and horseradish peroxidase (HRP) labelled secondary antibodies (1:2000; Santa Cruz Biotechnology).

### Gemcitabine treatment and collection of conditioned media

Colo-357 and MiaPaCa cells (1 × 10^6^/well) were seeded in 6-well plates and grown to subconfluence. Subsequently, cells were treated with either vehicle (PBS) or gemcitabine (10 μM) for 8 h. Media was replaced post-treatment with fresh low serum (2.5 %) containing media and cells were allowed to grow for next 48 h. Thereafter, conditioned media (CM) was collected, centrifuged for 10 min at 2500 rpm at 4°C, filtered with 0.22 μm membrane filter (EMD Millipore, Billerica, MA) to remove cell debris and designated as V-CM (from vehicle-treated cells) and Gem-CM (from gemcitabine-treated cells).

### *In vitro* cell growth assay

To examine the direct effect of gemcitabine on HUVECs growth, cells (1 × 10^4^ cells/well) were seeded in 96-well plate, incubated for 24 h, subsequently treated with various doses of gemcitabine (0–20 μM) for 48 h and growth was measured by WST-1 assay as described earlier [2, 41]. To examine the effect of V-CM or Gem-CM on HUVECs growth, cells (1 × 10^4^ cells/well) were seeded in 96-well plate and cultured for 24 h. Thereafter, media was replaced with V-CM or Gem-CM collected from Colo-357 and MiaPaCa cells and HUVECs were allowed to grow for next 24 or 48 h and cell growth was monitored as described above. To study the role of IL-8, Gem-CM was incubated with control IgG (200 ng/mL) or IL-8 neutralizing antibody (200 ng/mL) for 24 h and centrifuged prior to HUVECs treatment.

### Measurement of apoptosis

HUVECs were seeded (1 × 10^6^ cells/well) in 6-well plates and grown for 24 h. Subsequently, media was replaced with either V-CM or Gem-CM collected from Colo-357 and MiaPaCa cells. After 48 h incubation, HUVECs were harvested, stained with 7-Amino-Actinomycin (7-AAD) and PE Annexin V using commercially available kit and analyzed by flow cytometry as previously described [39].

### Cell cycle analysis

HUVECs were cultured in serum-free media for 48 h to synchronize them. Following synchronization, cells were treated with V-CM or Gem-CM for 24 h and processed for cell-cycle analysis as previously described by us [41, 42].

### RNA isolation and reverse transcription polymerase chain reaction (RT-PCR)

Total RNA was extracted using TRIzol reagent and complementary DNA (cDNA) was synthesized using 2 μg of total RNA and High-Capacity RNA-to-cDNA™ Kit. Quantitative real-time PCR (RT-PCR) was performed in 96-well plates using cDNA and SYBR Green Master Mix on an iCycler system (Bio-Rad, Hercules, CA) by specific sets of primer pairs (Supplementary Table 1). GAPDH was used as internal control. The thermal conditions for real-time PCR assays were as follows: cycle 1: 95°C for 10 min, cycle 2 (x 40): 95°C for 10 sec and 58°C for 45 sec.

### Western blot analysis

Protein extraction and immunoblotting was carried out as previously described [39, 43, 44] using anti-IL-8 antibody (1:1000) and HRP-labeled secondary antibodies (1:2000). β-actin served as a loading control. Immunodetection was performed following incubation of the immunoblots with SuperSignal West Femto Maximum sensitivity substrate. Protein bands were visualized using a LAS-3000 image analyzer (Fuji Photo Film Co., Tokyo, Japan).

### Enzyme-linked immunosorbent assay

Level of IL-8 in conditioned media obtained from vehicle- or gemcitabine-treated PC cells was measured by using human IL-8 ELISA kit as per manufacturer's instructions.

### *In vitro* capillary tube formation assay

Matrigel-coated 96 well plate was prepared according to manufacturer's instruction, and HUVECs (1 × 10^4^) were seeded in V-CM or Gem-CM or Gem-CM pre-incubated with control IgG /IL-8 neutralizing antibody (200 ng/mL) for 24 h. After 16 h of incubation, capillary like structure (CLS) formation was observed under the microscope and counted in ten random fields of view (100 X).

### Migration and invasion assays

Endothelial cells were plated on the top chamber of non-coated polyethylene teraphthalate membrane (2.5 × 10^5^ cells/inserts, for migration assay) or Matrigel-coated polycarbonate membrane (1 × 10^5^ cells/inserts, for invasion assay). V-CM or Gem-CM was added to the lower chamber as a chemo-attractant. After 16 h of incubation, cells on the upper surface of the insert membrane were removed with the help of cotton swab. Migrated or invaded cells to the bottom of the insert were fixed and stained with Diff-Quick cell staining kit and mounted on slide, random images were taken and plotted as average number of cells per field (200 X). To investigate the role of IL-8 in endothelial cells migration and invasion, we depleted IL-8 from Gem-CM by incubating it with control IgG or IL-8 neutralizing antibody (200 ng/mL) for 24 h and used it for migration and invasion assays. In parallel, HUVECs were pre-treated with conditioned media (V-CM or Gem-CM) for 12 h and effect on migration and invasion was examined as described above.

### Statistical analysis

All experiments were performed at least three times and data expressed as mean ± S.D. Wherever appropriate, the data were also subjected to unpaired two tailed Student's *t* test. *P* < 0.05 was considered statistically significant.

## SUPPLEMENTAL FIGURES AND TABLE



## References

[1] Siegel RL, Miller KD, Jemal A (2015). Cancer statistics. CA Cancer J Clin.

[2] Tyagi N, Bhardwaj A, Singh AP, McClellan S, Carter JE, Singh S (2014). p-21 activated kinase 4 promotes proliferation and survival of pancreatic cancer cells through AKT- and ERK-dependent activation of NF-kappaB pathway. Oncotarget.

[3] Arora S, Bhardwaj A, Singh S, Srivastava SK, McClellan S, Nirodi CS, Piazza GA, Grizzle WE, Owen LB, Singh AP (2013). An undesired effect of chemotherapy: gemcitabine promotes pancreatic cancer cell invasiveness through reactive oxygen species-dependent, nuclear factor kappaB- and hypoxia-inducible factor 1alpha-mediated up-regulation of CXCR4. J Biol Chem.

[4] Burris HA, Moore MJ, Andersen J, Green MR, Rothenberg ML, Modiano MR, Cripps MC, Portenoy RK, Storniolo AM, Tarassoff P, Nelson R, Dorr FA, Stephens CD (1997). Improvements in survival and clinical benefit with gemcitabine as first-line therapy for patients with advanced pancreas cancer: a randomized trial. J Clin Oncol.

[5] Moore MJ, Goldstein D, Hamm J, Figer A, Hecht JR, Gallinger S, Au HJ, Murawa P, Walde D, Wolff RA, Campos D, Lim R, Ding K (2007). Erlotinib plus gemcitabine compared with gemcitabine alone in patients with advanced pancreatic cancer: a phase III trial of the National Cancer Institute of Canada Clinical Trials Group. J Clin Oncol.

[6] Sun Y, Campisi J, Higano C, Beer TM, Porter P, Coleman I, True L, Nelson PS (2012). Treatment-induced damage to the tumor microenvironment promotes prostate cancer therapy resistance through WNT16B. Nat Med.

[7] Albini A, Tosetti F, Li VW, Noonan DM, Li WW (2012). Cancer prevention by targeting angiogenesis. Nat Rev Clin Oncol.

[8] Kerbel RS (2008). Tumor angiogenesis. N Engl J Med.

[9] Junttila MR, de Sauvage FJ (2013). Influence of tumour micro-environment heterogeneity on therapeutic response. Nature.

[10] Neesse A, Michl P, Frese KK, Feig C, Cook N, Jacobetz MA, Lolkema MP, Buchholz M, Olive KP, Gress TM, Tuveson DA (2011). Stromal biology and therapy in pancreatic cancer. Gut.

[11] Olive KP, Jacobetz MA, Davidson CJ, Gopinathan A, McIntyre D, Honess D, Madhu B, Goldgraben MA, Caldwell ME, Allard D, Frese KK, Denicola G, Feig C (2009). Inhibition of Hedgehog signaling enhances delivery of chemotherapy in a mouse model of pancreatic cancer. Science.

[12] Allison M (2012). Hedgehog hopes lifted by approval… and stung by failure. Nat Biotechnol.

[13] Michaelis M, Hinsch N, Michaelis UR, Rothweiler F, Simon T, ilhelm Doerr HW, Cinatl J, Cinatl J (2012). Chemotherapy-associated angiogenesis in neuroblastoma tumors. Am J Pathol.

[14] Shaked Y, Ciarrocchi A, Franco M, Lee CR, Man S, Cheung AM, Hicklin DJ, Chaplin D, Foster FS, Benezra R, Kerbel RS (2006). Therapy-induced acute recruitment of circulating endothelial progenitor cells to tumors. Science.

[15] Singh S, Wu S, Varney M, Singh AP, Singh RK (2011). CXCR1 and CXCR2 silencing modulates CXCL8-dependent endothelial cell proliferation, migration and capillary-like structure formation. Microvasc Res.

[16] Neufeld G, Kessler O (2006). Pro-angiogenic cytokines and their role in tumor angiogenesis. Cancer Metastasis Rev.

[17] Yoshida S, Ono M, Shono T, Izumi H, Ishibashi T, Suzuki H, Kuwano M (1997). Involvement of interleukin-8, vascular endothelial growth factor, and basic fibroblast growth factor in tumor necrosis factor alpha-dependent angiogenesis. Mol Cell Biol.

[18] Whipple C, Korc M (2008). Targeting angiogenesis in pancreatic cancer: rationale and pitfalls. Langenbecks Arch Surg.

[19] Stacker SA, Williams SP, Karnezis T, Shayan R, Fox SB, Achen MG (2014). Lymphangiogenesis and lymphatic vessel remodelling in cancer. Nat Rev Cancer.

[20] Kitadai Y (2010). Cancer-stromal cell interaction and tumor angiogenesis in gastric cancer. Cancer Microenviron.

[21] Matsuo Y, Ochi N, Sawai H, Yasuda A, Takahashi H, Funahashi H, Takeyama H, Tong Z, Guha S (2009). CXCL8/IL-8 and CXCL12/SDF-1alpha co-operatively promote invasiveness and angiogenesis in pancreatic cancer. Int J Cancer.

[22] Singh AP, Arora S, Bhardwaj A, Srivastava SK, Kadakia MP, Wang B, Grizzle WE, Owen LB, Singh S (2012). CXCL12/CXCR4 protein signaling axis induces sonic hedgehog expression in pancreatic cancer cells via extracellular regulated kinase- and Akt kinase-mediated activation of nuclear factor kappaB: implications for bidirectional tumor-stromal interactions. J Biol Chem.

[23] Fukuhara S, Zhang J, Yuge S, Ando K, Wakayama Y, Sakaue-Sawano A, Miyawaki A, Mochizuki N (2014). Visualizing the cell-cycle progression of endothelial cells in zebrafish. Dev Biol.

[24] Li A, Dubey S, Varney ML, Dave BJ, Singh RK (2003). IL-8 directly enhanced endothelial cell survival, proliferation, and matrix metalloproteinases production and regulated angiogenesis. J Immunol.

[25] Adams RH, Alitalo K (2007). Molecular regulation of angiogenesis and lymphangiogenesis. Nat Rev Mol Cell Biol.

[26] Folkman J, Klagsbrun M (1987). Angiogenic factors. Science.

[27] Chen Y, Shi M, Yu GZ, Qin XR, Jin G, Chen P, Zhu MH (2012). Interleukin-8, a promising predictor for prognosis of pancreatic cancer. World J Gastroenterol.

[28] Hidaka H, Ishiko T, Ishikawa S, Ikeda O, Mita S, Iwamura T, Chijiiwa K, Ogawa M (2005). Constitutive IL-8 expression in cancer cells is associated with mutation of p53. J Exp Clin Cancer Res.

[29] Xiao YC, Yang ZB, Cheng XS, Fang XB, Shen T, Xia CF, Liu P, Qian HH, Sun B, Yin ZF, Li YF (2015). CXCL8, overexpressed in colorectal cancer, enhances the resistance of colorectal cancer cells to anoikis. Cancer Lett.

[30] Yuan A, Chen JJ, Yao PL, Yang PC (2005). The role of interleukin-8 in cancer cells and microenvironment interaction. Front Biosci.

[31] Singh S, Sadanandam A, Nannuru KC, Varney ML, Mayer-Ezell R, Bond R, Singh RK (2009). Small-molecule antagonists for CXCR2 and CXCR1 inhibit human melanoma growth by decreasing tumor cell proliferation, survival, and angiogenesis. Clin Cancer Res.

[32] Singh S, Sadanandam A, Varney ML, Nannuru KC, Singh RK (2010). Small interfering RNA-mediated CXCR1 or CXCR2 knock-down inhibits melanoma tumor growth and invasion. Int J Cancer.

[33] Shin MR, Kang SK, Kim YS, Lee SY, Hong SC, Kim EC (2014). TNF-alpha and LPS activate angiogenesis via VEGF and SIRT1 signalling in human dental pulp cells. Int Endod J.

[34] Roebuck KA (1999). Regulation of interleukin-8 gene expression. J Interferon Cytokine Res.

[35] Sakamoto Y, Harada T, Horie S, Iba Y, Taniguchi F, Yoshida S, Iwabe T, Terakawa N (2003). Tumor necrosis factor-alpha-induced interleukin-8 (IL-8) expression in endometriotic stromal cells, probably through nuclear factor-kappa B activation: gonadotropin-releasing hormone agonist treatment reduced IL-8 expression. J Clin Endocrinol Metab.

[36] Xie K (2001). Interleukin-8 and human cancer biology. Cytokine Growth Factor Rev.

[37] Jung SK, Kim JH, Kim HJ, Ji YH, Kim JH, Son SW (2014). Silver nanoparticle-induced hMSC proliferation is associated with HIF-1alpha-mediated upregulation of IL-8 expression. J Invest Dermatol.

[38] Kunsch C, Rosen CA (1993). NF-kappa B subunit-specific regulation of the interleukin-8 promoter. Mol Cell Biol.

[39] Arora S, Bhardwaj A, Srivastava SK, Singh S, McClellan S, Wang B, Singh AP (2011). Honokiol arrests cell cycle, induces apoptosis, and potentiates the cytotoxic effect of gemcitabine in human pancreatic cancer cells. PLoS One.

[40] Osawa Y, Nagaki M, Banno Y, Brenner DA, Asano T, Nozawa Y, Moriwaki H, Nakashima S (2002). Tumor necrosis factor alpha-induced interleukin-8 production via NF-kappaB and phosphatidylinositol 3-kinase/Akt pathways inhibits cell apoptosis in human hepatocytes. Infect Immun.

[41] Bhardwaj A, Srivastava SK, Singh S, Arora S, Tyagi N, Andrews J, McClellan S, Carter JE, Singh AP (2014). CXCL12/CXCR4 signaling counteracts docetaxel-induced microtubule stabilization via p21-activated kinase 4-dependent activation of LIM domain kinase 1. Oncotarget.

[42] Srivastava SK, Bhardwaj A, Singh S, Arora S, McClellan S, Grizzle WE, Reed E, Singh AP (2012). Myb overexpression overrides androgen depletion-induced cell cycle arrest and apoptosis in prostate cancer cells, and confers aggressive malignant traits: potential role in castration resistance. Carcinogenesis.

[43] Bhardwaj A, Singh S, Srivastava SK, Honkanen RE, Reed E, Singh AP (2011). Modulation of protein phosphatase 2A activity alters androgen-independent growth of prostate cancer cells: therapeutic implications. Mol Cancer Ther.

[44] Bhardwaj A, Singh S, Srivastava SK, Arora S, Hyde SJ, Andrews J, Grizzle WE, Singh AP (2014). Restoration of PPP2CA expression reverses epithelial-to-mesenchymal transition and suppresses prostate tumour growth and metastasis in an orthotopic mouse model. Br J Cancer.

