# Supplementary material for: Gemcitabine triggers angiogenesis-promoting molecular signals in pancreatic cancer cells: Therapeutic implications
Source: Oncotarget. 2015 Apr 23;6(36):39140–50. doi: 10.18632/oncotarget.3784 (PMC4770762; doi:10.18632/oncotarget.3784)
Supplement: Supplementary file 1 [file oncotarget-06-39140-s001.pdf]

SUPPLEMENTAL FIGURES AND TABLE

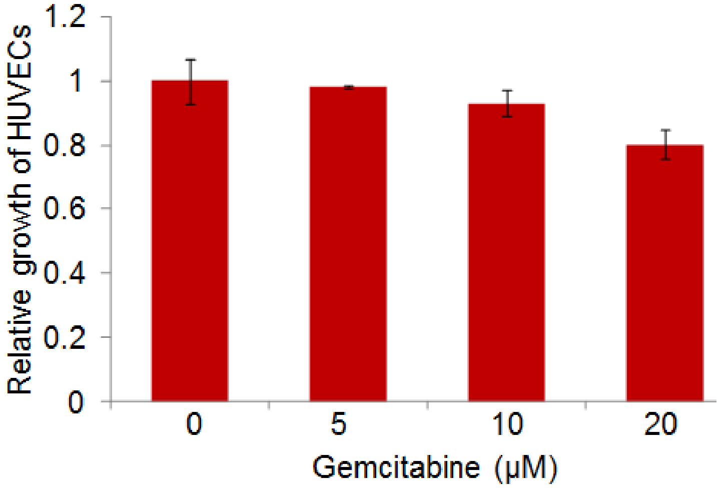

Supplementary Figure S1: Effect of gemcitabine on endothelial cells (HUVECs) growth. HUVECs ($1X10^4$ cells/well) were seeded in 96 well plate and treated with different concentrations of gemcitabine (0–20 µM) for 48 h and cell growth was monitored by WST-1 assay. Relative growth of HUVECs was calculated with respect to untreated control cells. Bars are expressed as mean ± SD; ($n = 3$).

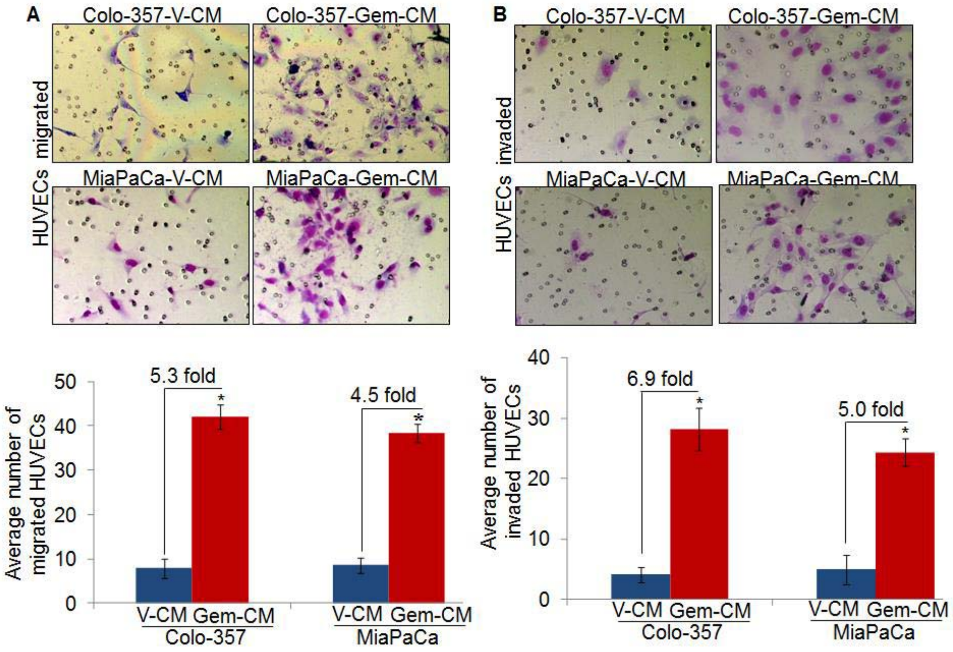

Supplementary Figure S2: Conditioned media from gemcitabine-treated pancreatic cancer cells increases the motility and invasiveness of HUVEC. Sub-confluent cultures of HUVEC were pre-treated with V-CM or Gem-CM and processed for **A.** migration and **B.** invasion assays as described earlier. V-CM or Gem-CM served as chemoattractant. After 16 h of incubation, migrated or invaded cells were fixed, stained and counted in 10 random view fields. Bars represent mean ± SD ($n = 3$) of number of migrated or invaded cells per field. *, $p < 0.05$.

Supplementary Table S1. Sequences of the primers used in the study

Primer's Name	Sequence
IFNB1-F	5'-ATGACCAACAAGTGTCTCCTCC-3'
IFNB1-R	5'- GGAATCCAAGCAAGTTGTAGCTC -3'
IL-8-F	5'-ACTGAGAGTGATTGAGAGTGGAC-3'
IL-8-R	5'-AACCCTCTGCACCCAGTTTTC-3'
TGFβ-F	5'- GGCCAGATCCTGTCCAAGC-3'
TGFβ-R	5'- GTGGGTTTCCACCATTAGCAC-3'
TRAIL-F	5'-TGCGTGCTGATCGTGATCTTC-3'
TRAIL-R	5'-GCTCGTTGGTAAAGTACACGTA-3'
PDGFA-F	5'- GCAAGACCAGGACGGTCATTT -3'
PDGFA-R	5'- GGCACTTGACACTGCTCGT -3'
TNF-α-F	5'- CCTCTCTCTAATCAGCCCTCTG -3'
TNF-α-R	5'- GAGGACCTGGGAGTAGATGAG -3'
IFN-γ-F	5'- TCGGTAACTGACTTGAATGTCCA -3'
IFN-γ-R	5'- TCGCTTCCCTGTTTTAGCTGC-3'
IL-1-F	5'- ATGATGGCTTATTACAGTGGCAA -3'
IL-1-R	5'- GTCGGAGATTCGTAGCTGGA -3'
IL-3-F	5'- CAGACAACGCCCTTGAAGACA -3'
IL-3-R	5'- GCCCTGTTGAATGCCTCCA -3'
IL-4-F	5'- CCAACTGCTTCCCCCTCTG -3'
IL-4-R	5'- TCTGTTACGGTCAACTCGGTG -3'
IL-5-F	5'- TGGAGCTGCCTACGTGTATG -3'
IL-5-R	5'- TCTGTTACGGTCAACTCGGTG -3'
IL-10-F	5'- TCAAGGCGCATGTGAACTCC-3'
IL-10-R	5'- GATGTCAAACTCACTCATGGCT-3'
CCL11-F	5'- CCCCTTCAGCGACTAGAGAG-3'
CCL11-R	5'- TCTTGGGGTCGGCACAGAT-3'
CXCL10-F	5'- GTGGCATTCAAGGAGTACCTC-3'
CXCL10-R	5'-TGATGGCCTTCGATTCTGGATT-3'
CCL2-F	5'-TCTGTGCCTGCTGCTCATAG-3'
CCL2-R	5'-GGGCATTGATTGCATCTGGC-3'
CXCL5-F	5'-AGCTGCGTTGCGTTTGTTTAC-3'
CXCL5-R	5'-TGGCGAACACTTGCAGATTAC-3'
CXCL6-F	5'-AGAGCTGCGTTGCACTTGTT-3'
CXCL6-R	5'-GCAGTTTACCAATCGTTTTGGGG-3'
CXCL1-F	5'-AGGCAGGGGAATGTATGTGC-3'
CXCL1-R	5'-GCCCCTTTGTTCTAAGCCAGA-3'

Primer's Name	Sequence
CXCL9-F	5'-CCAGTAGTGAGAAAGGGTCGC-3'
CXCL9-R	5'-AGGGCTTGGGGCAAATTGTT-3'
IFN-α1-F	5'-GCCTCGCCCTTTGCTTTACT-3'
IFN-α1-R	5'-CTGTGGGTCTCAGGGAGATCA-3'
MDK-F	5'-CGCGGTCGCCAAAAAGAAAG-3'
MDK-R	5'-TACTTGCAGTCGGCTCCAAAC-3'
ANGPT1-F	5'-AGAACCTTCAAGGCTTGGTTAC-3'
ANGPT1-R	5'-GGTGGTAGCTCTGTTTAATTGCT-3'
ANGPT2-F	5'-CTCGAATACGATGACTCGGTG-3'
ANGPT2-R	5'-TCATTAGCCACTGAGTGTTGTTT-3'
FGF2-F	5'-AGAAGAGCGACCCTCACATCA-3'
FGF2-R	5'-CGGTTAGCACACACTCCTTTG-3'
VEGF-F	5'-GAGGAGCAGTTACGGTCTGTG-3'
VEGF-R	5'-TCCTTTCCTTAGCTGACACTTGT-3'
GAPDH-F	5'-GGTGGTCTCCTCTGACTTCAACA-3'
GAPDH-R	5'-GTT GCTGTAGCCAAATTCGTTGT-3'
